# Resumption of Autophagy by Ubisol-Q_10_ in Presenilin-1 Mutated Fibroblasts and Transgenic AD Mice: Implications for Inhibition of Senescence and Neuroprotection

**DOI:** 10.1155/2019/7404815

**Published:** 2019-12-23

**Authors:** Caleb Vegh, Simon Pupulin, Darcy Wear, Lauren Culmone, Rachel Huggard, Dennis Ma, Siyaram Pandey

**Affiliations:** Department of Chemistry and Biochemistry, University of Windsor, Ontario, Canada

## Abstract

Alzheimer's disease (AD) is the most prevalent form of dementia and is associated with loss of memory, amyloid-beta plaque buildup, and neurofibrillary tangles. These features might be a result of neuronal cell death in the cerebral cortex and hippocampal regions of the brain. AD pathologies can be attributed to a variety of biochemical consequences including mitochondrial dysfunction, increased oxidative stress, and autophagy inhibition. Unfortunately, current therapeutics are limited only to symptomatic relief and do not halt the progression of neurodegeneration. Previous in vitro experiments have shown that a water-soluble formulation of coenzyme-Q_10_, Ubisol-Q_10_, can stabilize the mitochondria, prevent oxidative stress, and inhibit premature senescence in fibroblasts of AD patients. Since autophagy plays a critical role in maintenance and survival of neurons, we hypothesized that Ubisol-Q_10_ treatment could result in resumption of autophagy. Indeed, we observed induction of autophagy by Ubisol-Q_10_ treatment in AD fibroblasts as well as in the brains of transgenic AD mice. We found increased expression of autophagy-related genes beclin-1 and JNK1 following Ubisol-Q_10_ treatment of AD fibroblasts. These results were confirmed at the protein level by immunofluorescence and Western blotting. Interestingly, despite reduction of oxidative stress in cells due to Ubisol-Q_10_ treatment, autophagy inhibition leads to resumption of premature senescence in these PS-1 mutated fibroblasts indicating that autophagy is critical to prevent the senescence phenotype. Withdrawal of Ubisol-Q_10_ treatment also leads to the return of the senescence phenotype in AD fibroblasts indicating that constant supplementation of Ubisol-Q_10_ is required. Additionally, Ubisol-Q_10_ supplementation in the drinking water of double transgenic AD mice leads to increased expression of beclin-1 and JNK1 in the cortical region. Thus, the activation of autophagy by Ubisol-Q_10_ could be the mechanism for its ability to halt the progression of AD pathology in transgenic AD mice shown previously.

## 1. Introduction

Alzheimer's disease (AD) is the most common neurodegenerative disease and leading form of dementia across the globe. AD is characterized by decline in neurocognitive function leading to severe morbidity and eventually death [1]. While the majority of cases of AD are sporadic, mutations in the genes coding for amyloid precursor protein (APP) and presenilin-1 and presenilin-2 (PS-1 and PS-2) have been linked to familial and early-onset AD [2–4]. The exact etiology of AD is unknown, but some pathological features include the formation of toxic *β*-amyloid plaques and neurofibrillary tangles and neuron loss in the hippocampus [5, 6]. Furthermore, there are a number of mechanisms associated with AD which include the following: (1) increased oxidative stress [7], (2) mitochondrial dysfunction, and (3) impaired autophagy activity and accumulation of defective proteins/organelles [8]. Furthermore, it is hypothesized that these brain lesions/biochemical mechanisms occur before the symptoms of AD show implying that neuronal death is happening before disease diagnosis [9]. Current therapies only reduce the symptoms of AD, and there are unfortunately no treatments available that can prevent the progression of the disease. Many of the therapies used to treat symptoms of AD are chemomodulators, and extended use has shown to have toxic and adverse psychological side effects.

Neurons are virtually entirely dependent on oxidative phosphorylation for energy production, and as a result, mitochondrial dysfunction and oxidative stress are integral players in the development of AD pathology. Oxidative stress is a phenomenon where the amount of reactive oxygen species (ROS) in a cell increases as a result of reduced detoxification ability within the cell. It has been observed in patients with AD or mild cognitive impairment (MCI), where total antioxidant capacity (TOC) was reduced [10]. Importantly, it is proposed that increased ROS production precedes any other hallmarks of AD. The exact source of ROS in AD is unknown, but in some cases, it is known to originate from dysfunctional mitochondria [11–13]. Mutations in genes coding for components of cytochrome-c oxidase and presenilin-1 (PS-1) were found to be responsible for mitochondrial dysfunction in AD patients [13–15]. Increased mitochondrial dysfunction results in ineffective electron flow in the electron transport chain (ETC) leading to elevated ROS production. Neuronal cells are dependent on oxidative phosphorylation for energy production and thus are prone to produce more ROS. If the ROS quenching capabilities are reduced, then serious adverse effects occur due to ROS reacting with nucleic acids, proteins, and lipids [16]. Furthermore, increased oxidative stress also causes mitochondrial dysfunction that in turn produces more ROS thus creating a vicious cycle of increasing ROS and mitochondrial dysfunction [17]. This dysfunction has also been observed in peripheral tissue of AD patients including fibroblasts, making them suitable models for observing the biochemical pathology of AD [18]. Prolonged exposure of sublethal doses of ROS was shown to cause stress-induced premature senescence (SIPS) in AD fibroblasts. This phenomenon has been well characterized in fibroblasts with senescence-associated beta-galactosidase staining in fibroblasts from AD patients [8, 19].

As a consequence of increased oxidative stress, cells accumulate dysfunctional organelles and proteins that can lead to cellular dysfunction and apoptosis [16]. Cells have evolved efficient mechanisms to eliminate these damaged proteins/organelles using autophagy/proteasome degradation systems. Autophagy is the cell's mechanism for recycling old or damaged cytoplasmic constituents such as organelles or misfolded proteins [20]. There are multiple forms of autophagy, but the main form implicated in AD is macroautophagy. Interestingly, it has been shown that autophagy is either impaired or inhibited in AD patients [20–23]. Under these circumstances, defective/misfolded toxic proteins such as *β*-amyloid and dysfunctional organelles such as mitochondria can accumulate causing stress on cells leading to eventual cell death. Excessive oxidative stress can also affect various autophagy regulators such as beclin-1 (a major regulator of autophagosome maturation) [24]. Furthermore, PS-1 mutations have been shown to inhibit autophagy progression via blocking autophagosome maturation [25].

Based on these findings, oxidative stress, mitochondrial dysfunction, and autophagy could provide novel therapeutic targets for AD. By targeting these mechanisms, it would be possible to halt neurodegeneration in AD. Previously, we have observed that a water-soluble formulation of coenzyme-Q_10_ (Ubisol-Q_10_) has great potential to halt progression of neurodegenerative diseases including Parkinson's and Alzheimer's diseases [26]. Indeed, Ubisol-Q_10_ stabilized mitochondria and inhibited oxidative stress in vitro [27, 28]. Ubisol-Q_10_ also prevented oxidative SIPS and enhanced activation of autophagy via upregulation of beclin-1 (a major regulator of autophagy) in PS-1 mutated fibroblasts [8]. Additionally, Ubisol-Q_10_ at low doses (orally delivered) reduced circulating A*β* peptide, reduced oxidative stress, had positive effects on long-term and working memory, and drastically inhibited *β*-amyloid plaque formation in 16-month-old transgenic AD mouse brains [7].

Ubisol-Q_10_ seems to be a promising therapeutic for targeting AD pathology. Ubisol-Q_10_ not only is acting as a potent antioxidant but also could act as an activator of autophagy. PS-1 mutated fibroblasts could act as a good model for studying the induction of senescence and autophagy (under increased oxidative stress). Furthermore, the relationship between senescence and autophagy is not well understood. In this paper, we have shown that inhibition of oxidative stress by Ubisol-Q_10_ could not only inhibit SIPS but also activate autophagy. Here, we measured the differential gene expression profile of oxidative stress/autophagy genes in NHF and PSAF fibroblasts. Results indicated that Ubisol-Q_10_-treated PSAF cells display gene expression profiles similar to healthy NHF. In particular, there is upregulation of autophagy-related genes which was also confirmed at the protein level. Interestingly, inhibition of autophagy in Ubisol-Q_10_-treated PSAF cells leads to return of their SIPS phenotype. Furthermore, we demonstrated that autophagy is inhibited in the brains of transgenic AD mice and that it was activated with Ubisol-Q_10_ treatment. Thus, activation of autophagy is critical for the neuroprotective effect of Ubisol-Q_10_ in PSAF as well as in transgenic AD mice.

## 2. Materials and Methods

### 2.1. Cell Culture

Healthy nonfetal human skin fibroblasts (NHF) and PS-1 mutated AD familial type 3 fibroblasts (PSAF) from healthy and AD patients, respectively (Coriell Institute for Medical Research, Cat. Nos. AG09309 and AG04159, Camden, NJ, USA), were used throughout this study. NHF were derived from the skin of the toe, and PSAF were derived from the skin of the forearm. All fibroblasts were cultured in Eagle's minimum essential medium with Earle's salts and nonessential amino acids supplemented with 15% (*v*/*v*) fetal bovine serum (Thermo Scientific, Waltham, MA, USA) and 10 mg/mL gentamicin (Gibco BRL, VWR, Mississauga, ON, Canada). PSAF were grown in medium with or without supplementation with 50 *μ*g/mL Ubisol-Q_10_ (provided by Next™ Remedies, Toronto, ON, Canada) or with the PTS carrier. During the autophagy inhibition experiments mentioned below, another treatment group included PSAF that had Ubisol-Q_10_ withdrawn during the 48 hr period. All fibroblasts were grown at 37°C and 5% CO_2_.

### 2.2. Autophagy Inhibition

PSAF in the above-mentioned growth conditions were also subjected to autophagy inhibition via incubation with JNK1 inhibitor SP600125. SP600125 is a well-known inhibitor of autophagy via beclin-1 inhibition as JNK1 is a major regulator of beclin-1 activation. PSAF were incubated for 48 hrs in media containing 10 *μ*M SP600125 (Sigma-Aldrich, Oakville, ON, Canada, Cat. No. S5567) and 0.1% DMSO to maintain solubility.

### 2.3. Quantitative Polymerase Chain Reaction (qPCR) of Autophagy and Oxidative Stress-Related Genes

A RT^2^ profile PCR assay was performed in order to measure the relative gene expression of autophagy and oxidative stress-related genes. RNA from cells was extracted using the Qiagen RNeasy Mini Kit (Qiagen Inc., Toronto, ON, Canada, Cat. No. 74106). RNA quality and quantity were determined my measuring the A280:A260 (Nanodrop 200). cDNA was produced from RNA extracts using a RT^2^ First Strand Kit (Qiagen Inc., Toronto, ON, Canada, Cat. No. 330401). Following cDNA synthesis, qPCR was performed on samples using the RT^2^ Profiler PCR Array Human Oxidative Stress Plus Array (Qiagen Inc., Toronto, ON, Canada, Cat. No. PAHS-065Y). The array containing 84 primers that probe for autophagy and oxidative stress-related genes was performed following the manufacturer's protocol using SYBR Green. Real-time amplification data was acquired using the ABI ViiA™ 7 real-time PCR system with a 384-well block and respective ABI ViiA™ 7 software. Amplification occurred for 40 cycles for 15 s at 95°C and 1 min at 60°C. A melting curve of each sample confirmed specificity of amplification, and gene expression was normalized to housekeeping genes. Results were obtained as fold changes in gene expression between the controls and treated groups using the *ΔΔ*CT method.

### 2.4. Measurement of Reactive Oxygen Species (ROS)

ROS production was measured by membrane permeable 2′-7′-dichlorofluorescein diacetate (H_2_DCFDA) (Life Technologies Inc., Cat. No. D-399, Burlington, ON, Canada) which is oxidized by ROS to fluorescent 2′,7′-dichlorofluorescein (DCF) following cleavage of acetate groups by intracellular esterases. Cells were incubated in 10 *μ*M H_2_DCFDA dissolved in DMSO for 30 min at 37°C. DCF fluorescence was detected using epifluorescence microscopy via a Leica DMI6000 B inverted microscope (Leica Microsystems, Concord, ON, Canada). Fluorescence was quantified in images captured using ImageJ software.

### 2.5. Senescence-Associated Beta-Galactosidase (SA-*β*-gal) Staining

SA-*β*-gal stain was used to detect prematurely senescent fibroblasts. Cells were washed in 1x PBS, fixed for 4 min at room temperature in 3% formaldehyde, washed with 1x phosphate-buffered saline (PBS) again, and incubated at 37°C with no CO_2_ with fresh SA-*β*-gal staining solution (1 mg/mL X-Gal, 20 mg/mL dimethylformamide, 40 mM citric acid, 40 mM sodium phosphate, 5 mM potassium ferrocyanide, 5 mM potassium ferricyanide, 150 mM NaCl, and 2 mM MgCl_2_, pH 6.0) for 16 hrs. Senescent cells were detected using phase contrast microscopy via a Leica DMI6000 B inverted microscope (Leica Microsystems, Concord, ON, Canada). The proportion of cells staining positive for SA-*β*-gal activity was counted manually.

### 2.6. Monodansylcadaverine (MDC) Staining for Autophagic Vacuoles

Cells were seeded on 4 chamber slides (Bio Basic Inc., Markham, ON, Canada, Cat. No. SP41215) 24 hrs prior to experimentation. Cells were incubated with 0.1 mM MDC (Sigma-Aldrich, Canada, Cat. No. 30432, Mississauga, ON, Canada) dissolved in DMSO for 15 min. Cells were washed with PBS, and cells containing autophagic vacuoles tagged with MDC were detected using epifluorescence microscopy via a Leica DMI6000 B inverted microscope (Leica Microsystems, Concord, ON, Canada). Fluorescence was quantified in images captured using ImageJ software.

### 2.7. Immunofluorescence Staining

Cells were seeded on 4 chamber slides (Bio Basic Inc., Markham, ON, Canada, Cat. No. SP41215) 24 hrs prior to experimentation. Cells were fixed with 3.7% formaldehyde at room temperature, followed by permeabilization with 0.15% Triton X-100 for 2 minutes, and then blocked with 5% bovine serum albumin (BSA) for 1 hr at room temperature. Cells were incubated for 1 hr at room temperature in the following primary antibodies: beclin-1 (mouse IgG, 1 : 500, Cat. No. sc-48342), C-Jun terminal kinase 1 (JNK1) (mouse IgG, 1 : 500, Cat. No. sc-137018), p21 (mouse IgG, 1 : 250, Cat. No. sc-817) (Santa Cruz Biotechnologies), 4-hydroxynonenal (rabbit IgG, 1 : 500, Cat. No. ab46545), and LC3B (rabbit IgG, 1 : 500, Cat No. ab192890) (Abcam Inc.). Cells were washed with PBS and incubated with horse anti-mouse FITC (1 : 500, MJS BioLynx Inc., Cat. No. Fl-2000) and/or a goat anti-rabbit Alexa Fluor™ 568 (1 : 500, Thermo Scientific Canada, Cat. No. A11011) secondary antibody for 1 hr at room temperature. Cells were washed again with PBS and incubated with 10 *μ*M Hoechst 33342 (Molecular Probes, Eugene, OR, USA Cat. No. H3570). Cells were imaged using epifluorescence microscopy via a Leica DMI6000 B inverted microscope (Leica Microsystems, Concord, ON, Canada). Fluorescence was quantified in images captured using ImageJ software.

### 2.8. Western Blot Analyses

Sodium dodecyl sulfate-polyacrylamide gel electrophoresis (SDS-PAGE) was performed on protein samples from fibroblasts and then transferred onto a polyvinylidene difluoride (PVDF) membrane. The membrane was blocked with 5% *w*/*v* milk TBST (tris-buffered saline Tween-20) solution for 1 hr. Membranes were incubated in mouse anti-beclin-1 IgG (1 : 1000, Santa Cruz Biotechnology Inc., Mississauga, ON, Canada, Cat. No. sc-48342) overnight at 4°C. Membranes were washed with TBST, incubated in goat anti-mouse horseradish peroxidase-conjugated secondary IgG (1 : 2000, Novus Biologicals, Oakville, ON, Canada, Cat. No. NBP2-30347H) for 1 hr at room temperature, washed with TBST, and imaged for bands with a chemiluminescence reagent (Thermo Scientific Canada, Cat. No. 34095). Densitometric analysis was performed using ImageJ software.

### 2.9. Animal Care

The same protocol was performed according to Muthukumaran et al. 2018. Experiments performed on animals were approved by the University of Windsor's Animal Care Committee in accordance with the Canadian Council of Animal Care guidelines. Twelve male double transgenic APP/PS-1 mice (Jackson Laboratory; strain: B6C3-Tg(APPswe,PSEN1dE9)85Dbo/Mmjax) and six male C57BL/6 wild-type counterpart mice (Charles River Laboratories) were housed in groups of three or four. Transgenic mice were housed separately to avoid any social hierarchies due to functional neurological changes. The home cages contained baby-food jars, overturned cardboard cup holders, and cardboard tubes to provide environmental enrichment. Mice had continuous access to food and water, and their weight was measured once a week. The colony room was maintained at 20°C, and mice were under a controlled 12 hr : 12 hr dark-light cycle. Following the experimental period, the mice (approximately 18 months old) were euthanized and perfused using ice-cold PBS containing 28 *μ*g/mL heparin (Sigma-Aldrich, Canada, Cat. No. H3393) followed by tissue fixation with ice-cold 10% formaldehyde made in PBS.

### 2.10. Animal Treatment Regimen

The treatment group consisted of Ubisol-Q10 (Zymes LLC, Hasbrouck, NJ, USA)-supplemented drinking water at a concentration of 200 *μ*g/mL which contained an equivalent of 50 *μ*g/mL. The control groups consisted of either water supplemented with the PTS carrier molecule (Zymes LLC, Hasbrouck, NJ, USA) or regular drinking water. Fresh water was provided weekly, and the treatment period lasted 18 months.

### 2.11. Immunohistochemistry

Following perfusion, brains were extracted and stored in 10% formalin at 4°C. Brains were transferred to 30% (*w*/*v*) sucrose (made in 1x PBS) prior to sectioning. Once brains sank in 30% sucrose, they were cryosectioned at 30 *μ*m thickness with Shandon™ M-1 embedding matrix (Thermo Scientific Canada, Cat. No. 1310TS) onto glass microscope slides. Slides were washed twice with tris-buffered saline (TBS) for 5 min each followed by incubation with 1% H_2_O_2_ to block endogenous peroxidases. Slides were rinsed twice with TBS for 5 min each followed by a 30 min block using a DAKO serum-free protein block (Agilent Technologies Canada Inc., Cat. No. X0909) and normal serum according to instructions of the Vector Laboratories Vectastain Elite ABC-Peroxidase kit, mouse IgG (MJS BioLynx Inc., Cat. No. VECTPK6102). Following blocking, sections were incubated overnight at 4°C in the following primary antibodies: beclin-1 (mouse IgG, 1 : 500, Cat. No. sc-48342) and C-Jun terminal kinase 1 (JNK1) (mouse IgG, 1 : 500, Cat. No. sc-137018) (Santa Cruz Biotechnologies). The slides were washed twice with TBS for 5 min followed by incubation of secondary biotinylated antibody according to instructions from the Vectastain Elite ABC-Peroxidase kit. Slides were washed again with TBS, and then, tissue sections were incubated with avidin-conjugated horseradish peroxidase from the Vectastain Elite ABC-Peroxidase kit for 45 min. Slides were washed with TBS and incubated with 3,3′-diaminobenzidine (DAB) stain solution according to the Vector Laboratories DAB peroxidase substrate kit (MJS BioLynx Inc., Cat. No. SK-4100). Sections were dehydrated with two 5 min anhydrous ethanol washes and a 7 min xylene wash followed by cover slipping using Permount® mounting medium (Fisher Scientific Canada, Cat. No. SP15-500). Cells were imaged using bright-field microscopy via a Leica DMI6000 B inverted microscope (Leica Microsystems, Concord, ON, Canada).

## 3. Results

### 3.1. Autophagy-Related Gene/Protein Expression Was Enhanced in PS-1 Mutated Alzheimer's Disease Fibroblasts (PSAF) Treated with Ubisol-Q_10_

**Figure 1:**
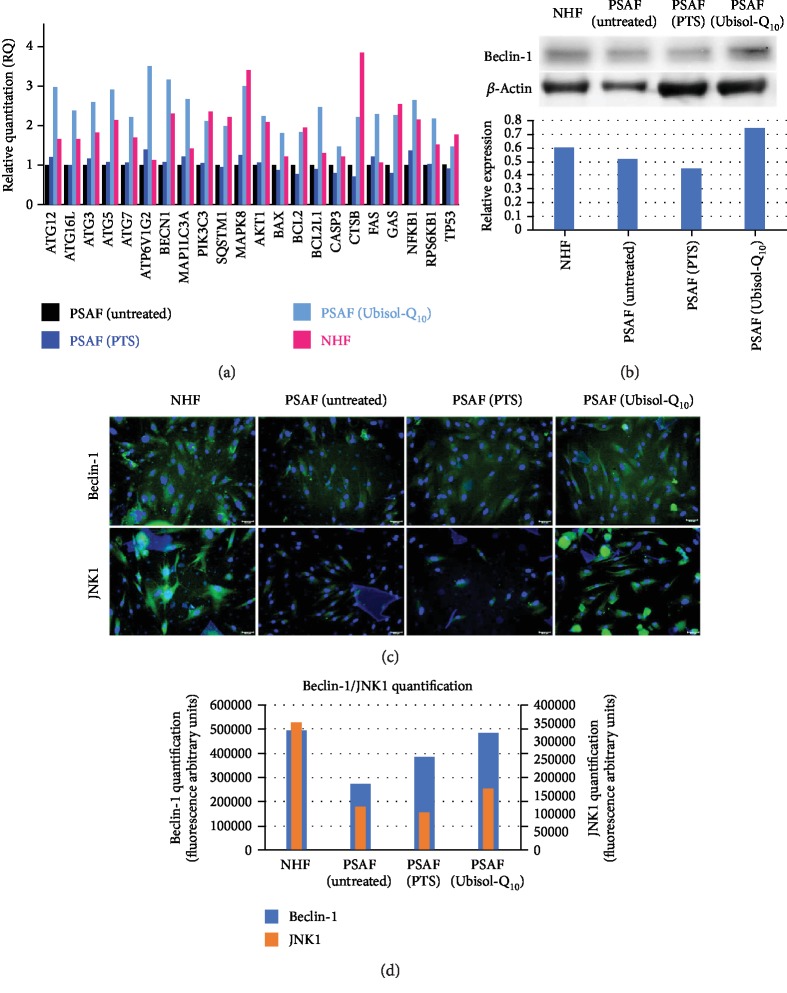
(a) Autophagy-related gene expression profile for Normal Human Fibroblasts (NHF), untreated PSAF, PTS-treated PSAF, and Ubisol-Q10-treated PSAF. NHF cells served as a positive control whereas untreated and PTS-treated AD cells served as negative controls. Notably, autophagy-related genes beclin-1, MAPK8/JNK1, and CTSB were upregulated in AD cells treated with Ubisol-Q10 bringing expression levels similar to or higher than NHF. (b) Beclin-1 probing of whole cell lysates via Western blot from NHF and PSAF. Supporting gene analysis, Beclin-1 was upregulated in Ubisol-Q10-treated PSAF and not untreated or PTS-treated AD cells. (c, d) Immunofluorescence staining of NHF and PSAF probing for beclin-1 (green) and JNK1 (green) and quantification of fluorescence, respectively. Treatment of PSAF cells with Ubisol-Q10 led to increased staining for beclin-1 and JNK1 compared to the untreated and PTS-treated groups indicating upregulation of autophagic proteins. These Ubisol-Q10-treated PSAF were stained in a comparable manner to NHF cells. Nuclei were counterstained with Hoechst for visualization. Micrographs were taken at 200x magnification. Scale bar = 50 *μ*m.

Mutations in PS-1 have been shown to reduce progression of autophagy leading to buildup of dysfunctional mitochondria and generation of increased ROS [11, 12, 25]. We compared expression levels of genes associated with autophagy and oxidative stress between NHF, untreated PSAF, and PSAF treated with either PTS (placebo/vehicle) or Ubisol-Q_10_ (Figure 1(a)). The gene expression profile of PSAF treated with Ubisol-Q_10_ was similar to that of NHF. Untreated PSAF and PSAF treated with PTS had lower overall expression levels of genes associated with autophagy/oxidative stress compared with PSAF treated with Ubisol-Q_10_ or NHF. In particular, beclin-1 (a major autophagy regulator) expression was enhanced in Ubisol-Q_10_-treated PSAF compared to untreated PSAF. Ubisol-Q_10_ caused enhancement of MAPK8/JNK1 (a major activator of beclin-1) expression at a similar level to that of healthy NHF. Confirming gene expression profiling results, Western blotting indicated increased expression of proteins beclin-1 and JNK1 in Ubisol-Q_10_-treated PSAF similar to NHF whereas untreated PSAF or the ones given PTS had significantly reduced expression of beclin-1 and JNK1 protein (Figures 1(b)–1(d)).

### 3.2. Inhibition of Autophagy by SP600125 in Ubisol-Q_10_-Treated PSAF Leads to Return of SIPS Phenotype

**Figure 2:**
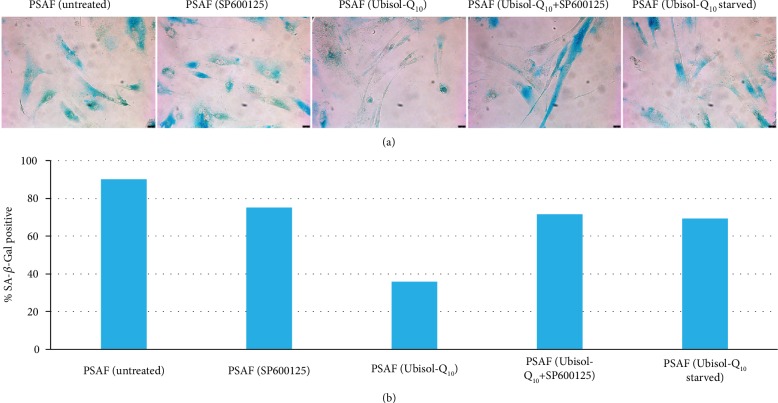
Ubisol-Q_10_-treated PSAF incubated with SP600125 JNK1 inhibitor showed a resumption of premature senescence bringing fibroblasts back to the original AD morphology. Cells were incubated in senescence-associated beta-galactosidase to identify senescent fibroblasts. Micrographs were taken at 200x. Scale bar = 25 *μ*m.

It was previously reported that oxidative stress-induced premature senescence was prevented in PSAF in the presence of Ubisol-Q_10_ [8]. We investigated the role of autophagy in preventing SIPS in PSAF treated with Ubisol-Q_10_ by treating them with SP600126, a well-known inhibitor of autophagy by blocking JNK1, a major activator of beclin-1 [29, 30]. Confirming previous results, the relative amount of cells staining positive for blue SA-*β*-gal was reduced in Ubisol-Q_10_-treated cells compared to untreated PSAF. The proportion of cells staining positive for blue SA-*β*-gal in PSAF treated with Ubisol-Q_10_ and SP600125 was increased compared to that of Ubisol-Q_10_ PSAF not incubated with SP600125 (Figure 2). A similar observation was made when previously treated PSAF had Ubisol-Q_10_ treatment withdrawal for 48 hrs in which staining for blue SA-*β*-gal increased. There was little to no observable difference in SA-*β*-gal staining between untreated PSAF and untreated PSAF incubated in SP600125.

**Figure 3:**
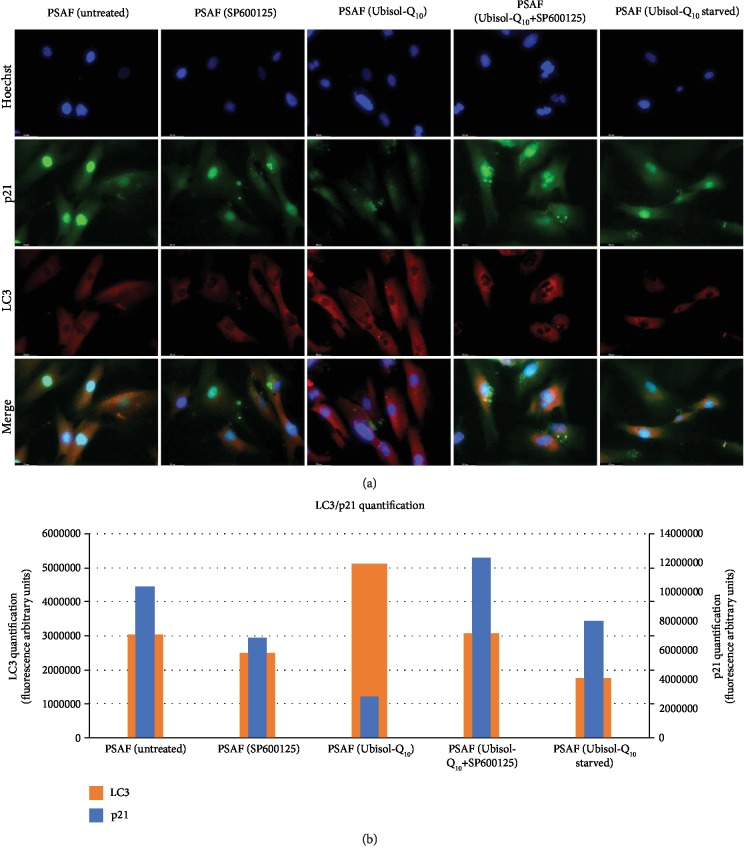
Treatment with SP600125 leads to resumption of senescence phenotype and reduced autophagosome formation in Ubisol-Q_10_-treated cells. (a, b) Immunofluorescence staining of PSAF probing for p21 (green) and LC3 (red) and quantification of fluorescence, respectively. Treatment of PSAF with Ubisol-Q_10_ leads to increased staining for LC3 puncta an autophagosome indicator. Ubisol-Q_10_ also lead to reduced staining for p21, an indicator of senescence. Ubisol-Q_10_-treated PSAF incubated in the presence of SP600125 resulted in increased staining for p21 and reduced staining for LC3 puncta similar to that of untreated PSAF, PSAF treated with SP600125, and PSAF starved of Ubisol-Q_10_ for 48 hours. Nuclei were counterstained with Hoechst for visualization. Micrographs were taken at 400x magnification. Scale bar = 50 *μ*m.

In the presence of cell stressors such as ROS generation, p21 is known to promote cell cycle arrest. Previously, it was shown that PSAF had elevated expression of p21 compared to Ubisol-Q_10_-treated PSAF [8]. Indeed, the same observation was made when cells were probed for p21 via immunofluorescence (Figures 3(a) and 3(b)). PSAF treated with Ubisol-Q_10_ showed minimal staining for p21 compared to untreated PSAF and PSAF incubated in SP600125. In the presence of SP600125, Ubisol-Q_10_-treated cells showed increased expression similar to untreated PSAF. Similarly, when Ubisol-Q_10_-treated PSAF were starved of Ubisol-Q_10_ after 48 hours, p21 expression increased in a comparable manner similar to untreated PSAF.

### 3.3. Ubisol-Q_10_ Treatment Withdrawal or Treatment with Autophagy Inhibitor SP600125 Results in Reduced Autophagosome Formation without Affecting Endogenous Levels of ROS

**Figure 4:**
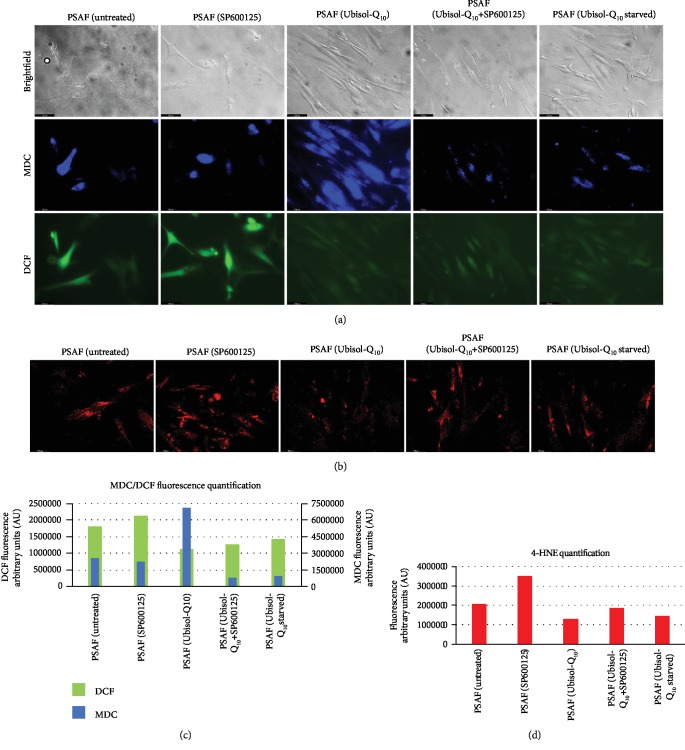
(a, c) Inhibition of autophagy via SP600125 leads to reduced autophagosome formation (blue) and an increase in oxidative stress (green) in PSAF following treatment with Ubisol-Q_10_. PSAF starved of Ubisol-Q_10_ returned to AD morphology. Cells were incubated with monodansylcadaverine (MDC) to visualize autophagic vacuoles. Cells were also incubated with 2′,7′-dichlorofluorescein diacetate which is oxidized to fluorescent 2′,7′-dichlorofluorescein (DCF) for visualization of reactive oxidative species (ROS) production. (b, d) Similarly to DCF, immunofluorescence staining for 4-hydroxynonenal (4-HNE) a peroxidized lipid and an oxidative stress indicator was reduced in Ubisol-Q_10_-treated PSAF. Ubisol-Q_10_-treated PSAF incubated in SP600125 and PSAF starved of Ubisol-Q_10_ for 48 hours showed increased staining for 4-HNE similar to untreated PSAF. Micrographs were taken at 200x. Scale bar = 100 *μ*m.

In a past study, generation of endogenous ROS was shown to be elevated in untreated PSAF compared to NHF [8]. Interestingly, the opposite observation was made for autophagosome formation (as demonstrated by MDC staining for autophagosomes). We investigated the effect autophagy inhibition has on autophagosome formation and endogenous ROS levels (Figure 4). Untreated PSAF or the ones given Ubisol-Q_10_ were incubated with autophagy inhibitor SP600125, and the presence of autophagosome formation and endogenous ROS was observed using MDC and DCF fluorescence staining, respectively.

Confirming results from the previous study, PSAF treated with Ubisol-Q_10_ had significantly higher levels of staining for MDC compared to untreated PSAF, indicating increased autophagosome formation (Figure 4). Ubisol-Q_10_-treated cells incubated with SP600125 had reduced autophagosome formation compared to those incubated without SP600125 as indicated by reduced proportion of cells staining positive for MDC. Similarly, when Ubisol-Q_10_ treatment was withdrawn, autophagosome formation decreased. Untreated PSAF incubated in SP600125 showed little to no observable difference in MDC staining compared to untreated PSAF not incubated in SP600125. Another method to measure autophagosome formation and overall autophagic flux is the detection of LC3 puncta [31]. Similar to MDC staining, LC3 puncta was increased in Ubisol-Q_10_-treated PSAF compared to untreated PSAF, PSAF incubated in SP600125, and Ubisol-Q_10_-starved cells (Figures 3(a) and 3(b)). In the presence of SP600125, LC3 puncta was reduced in PSAF treated with Ubisol-Q_10_.

Similar to results in the previous study mentioned above, endogenous levels of ROS were reduced in PSAF given Ubisol-Q_10_ compared to untreated PSAF (Figure 4). Ubisol-Q_10_-treated PSAF incubated with SP600125 or PSAF with withdrawn Ubisol-Q_10_ treatment showed minor increases in levels of endogenous ROS compared to the ones constantly given Ubisol-Q_10_ and not incubated in SP600125. Similar to DCF staining, 4-hydroxynonenal a lipid peroxidation by-product and indicator of oxidative stress was reduced in cells given Ubisol-Q_10_. Ubisol-Q_10_-treated PSAF incubated with SP600125 or PSAF with withdrawn Ubisol-Q_10_ treatment showed slightly elevated levels of endogenous ROS compared to the ones constantly given Ubisol-Q_10_ and not incubated in SP600125 (Figures 4(b) and 4(d)).

### 3.4. Ubisol-Q_10_ Treatment Leads to Increased Expression of Autophagy-Related Proteins In Vivo

**Figure 5:**
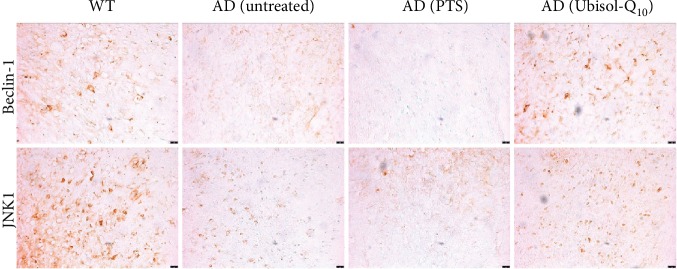
Immunohistochemical staining of the cerebral cortex from double transgenic mice probing for beclin-1 and JNK1. Oral supplementation of Ubisol-Q_10_ results in the upregulation of beclin-1 and JNK1 in transgenic mice similar to wild-type mice as indicated by the increased immunoactivity in the Ubisol-Q_10_-treated AD group compared to the untreated and PTS-treated groups. Micrographs were taken at 100x. Scale bar = 25 *μ*m.

As mentioned above, we observed an increase in expression of autophagic proteins beclin-1 and JNK1 in PSAF treated with Ubisol-Q_10_ similar to NHF. We investigated if these same proteins are upregulated in double transgenic AD mice treated with Ubisol-Q_10_. Similar to PSAF, these double transgenic mice contain a PS-1 mutation [25]. Previously, it has been shown that oral feeding of Ubisol-Q_10_ to these mice ameliorated AD pathology [7]. The brain tissues of these same mice were analyzed using immunostaining (Figure 5). Indeed, beclin-1 and JNK1 were both upregulated in transgenic mice given drinking water supplemented with Ubisol-Q_10_ similar to the wild-type mice. Transgenic mice given unsupplemented or PTS-supplemented drinking water had significantly reduced expression of beclin-1 and JNK1 compared to wild-type mice or Ubisol-Q_10_-treated mice.

## 4. Discussion

In this report, we have shown that activation of autophagy is critical for cellular health. We have demonstrated that presenilin-1 mutations lead to inhibition of autophagy in PSAF as well as the brains of transgenic AD mice. Importantly, Ubisol-Q_10_ lead to resumption of autophagy and inhibition of senescence in PSAF. Furthermore, resumption of autophagy was also observed in Ubisol-Q_10_-treated transgenic AD mice. Thus, treatment with Ubisol-Q_10_ leads to amelioration of adverse effects of PS-1 mutation in vitro and in vivo by activation autophagy.

Presenilin-1 mutations are one of the major causes of early-onset AD. Fibroblasts from AD patients containing a PS-1 mutation as well as those from age-matched healthy individuals are easily available. The detrimental effect of PS-1 mutations has been studied in PSAF [8]. These cells undergo premature senescence earlier (around 12 population doublings) compared to healthy fibroblasts which undergo senescence around 40 population doublings. PSAF are under constant elevated levels of oxidative stress. Generally, when cells are faced with increased oxidative stress, autophagy is triggered as a prosurvival response. It is hypothesized that if autophagy is unable to progress, cells would undergo senescence. Ubisol-Q_10_ a water-soluble formulation of coenzyme-Q_10_ has been shown to protect neuronal cells from oxidative stress and excitotoxicity [28, 32]. Interestingly, Ubisol-Q_10_ treatment prevented oxidative stress and SIPS in PSAF and enhanced autophagy [8]. Indeed, when we compared the gene expression of oxidative stress/autophagy-related genes in NHF, PSAF, and Ubisol-Q_10_-treated PSAF, we found that several autophagy-related genes were upregulated in Ubisol-Q_10_ PSAF. The gene expression pattern of Ubisol-Q_10_-treated PSAF was found similar to that of healthy NHF (Figure 1(a)). These results indicate that Ubisol-Q_10_ treatment of PSAF enables these cells to overcome the deleterious effects of the PS-1 mutation. Expression of some of these autophagy-related genes was confirmed at the protein level by Western blotting and immunofluorescence (Figures 1(b) and 1(c)). Indeed, this was indicated by the increased expression of beclin-1 via Western blotting and immunofluorescence in Ubisol-Q_10_-treated PSAF. Interestingly, beclin-1 was expressed in higher amounts in Ubisol-Q_10_-treated PSAF compared to NHF. Furthermore, JNK1 a major activator of beclin-1 was also upregulated in Ubisol-Q_10_-treated PSAF comparable to NHF. These observations indicate that Ubisol-Q_10_ treatment could be triggering autophagy and inhibiting senescence at the same time. The question proposed here is whether autophagy is preventing PSAF from undergoing SIPS, and inhibiting autophagy could result in the return of the SIPS phenotype in PSAF. Indeed, when Ubisol-Q_10_-treated PSAF were treated with autophagy inhibitor SP600125 (a known inhibitor of beclin-1 via JNK1 inhibition [29, 30]), we saw a drastic decrease in autophagosome formation (Figures 4(a), 4(c), and 3) as well as the return of the SIPS phenotype (Figures 2, 3(a), and 3(b)). It should also be noted that when Ubisol-Q_10_ treatment was withdrawn, autophagosome formation decreased and the SIPS phenotype returned in PSAF indicating that constant treatment with Ubisol-Q_10_ is required to maintain a healthy cell morphology (Figures 2 and 4(a)). Another important observation was that despite reduced ROS production (Figure 4), withdrawal of Ubisol-Q_10_ resulted in reduction of autophagy and resumption of senescence (Figures 2 and 3). This could indicate that Ubisol-Q_10_-induced autophagy is independent of antioxidative effects. Thus, it seems that stress (such as oxidative stress, DNA damage, starvation, and mitochondrial dysfunction)-induced autophagy and SIPS have an inverse relationship and could play a very important role in homeostasis and maintenance of neuronal cells.

Mitochondrial dysfunction has been shown to be involved in autophagy, senescence, and apoptosis by several researchers [33]. Previously, Ubisol-Q_10_ has been shown to inhibit bax-induced destabilization of mitochondria in mammalian cells [34]. Thus, it seems that Ubisol-Q_10_ has the potential to prevent the deleterious effects of PS-1 mutations and reverse the SIPS phenotype by resumption of autophagy in PSAF. Most importantly, Ubisol-Q_10_ treatment has demonstrated very clear amelioration of AD pathology in double transgenic mice containing mutated PS-1 and amyloid precursor protein (APP) [7]. Could this effect be the result of activation of autophagy via Ubisol-Q_10_ in these mice? When we used brain sections from the mice of the same experiment and stained for autophagy-related proteins, we observed upregulation of beclin-1 and JNK1 in the cortex of Ubisol-Q_10_-treated mice appearing similar to brains of wild-type mice (Figure 5). This is extremely important as Ubisol-Q_10_, a simple/GRAS approved nutritional supplement, has shown unprecedented activation of autophagy leading to reversal of SIPS in vitro and halting of the progression of AD pathology in vivo. Furthermore, this is a nutritional supplement that can be taken perpetually without any side effects.

## Data Availability

The data from this work is available.

## References

[1] McKhann G., Knopman D. S., Chertkow H. (2011). The diagnosis of dementia due to Alzheimer's disease: recommendations from the National Institute on Aging-Alzheimer's Association workgroups on diagnostic guidelines for Alzheimer's disease. *Alzheimer's & Dementia*.

[2] Murrell J., Farlow M., Ghetti B., Benson M. D. (1991). A mutation in the amyloid precursor protein associated with hereditary Alzheimer’s disease. *Science*.

[3] Sherrington R., Rogaev E. I., Liang Y. (1995). Cloning of a gene bearing missense mutations in early-onset familial Alzheimer’s disease. *Nature*.

[4] Levy-Lahad E., Wasco W., Poorkaj P. (1995). Candidate gene for the chromosome 1 familial Alzheimer’s disease locus. *Science*.

[5] Rhein V., Eckert A. (2007). Effects of Alzheimer’s amyloid-beta and tau protein on mitochondrial function -- role of glucose metabolism and insulin signalling. *Archives of Physiology and Biochemistry*.

[6] Huang Y., Liu X. Q., Wyss-Coray T., Brecht W. J., Sanan D. A., Mahley R. W. (2001). Apolipoprotein E fragments present in Alzheimer’s disease brains induce neurofibrillary tangle-like intracellular inclusions in neurons. *Proceedings of the National Academy of Sciences of the United States of America*.

[7] Muthukumaran K., Kanwar A., Vegh C. (2017). Ubisol-Q10 (a nanomicellar water-soluble formulation of CoQ10) treatment inhibits Alzheimer-type behavioral and pathological symptoms in a double transgenic mouse (TgAPEswe, PSEN1dE9) model of Alzheimer’s disease. *Journal of Alzheimer's Disease*.

[8] Ma D., Stokes K., Mahngar K., Domazet-Damjanov D., Sikorska M., Pandey S. (2014). Inhibition of stress induced premature senescence in presenilin-1 mutated cells with water soluble coenzyme Q_10_. *Mitochondrion*.

[9] Savva G. M., Wharton S. B., Ince P. G., Forster G., Matthews F. E., Brayne C. (2009). Age, neuropathology, and dementia. *The New England Journal of Medicine*.

[10] Guidi I., Galimberti D., Lonati S. (2006). Oxidative imbalance in patients with mild cognitive impairment and Alzheimer's disease. *Neurobiology of Aging*.

[11] Bonda D. J., Wang X., Perry G. (2010). Oxidative stress in Alzheimer disease: a possibility for prevention. *Neuropharmacology*.

[12] Swerdlow R. H., Burns J. M., Khan S. M. (2014). The Alzheimer’s disease mitochondrial cascade hypothesis: progress and perspectives. *Biochimica et Biophysica Acta*.

[13] Davis R. E., Miller S., Herrnstadt C. (1997). Mutations in mitochondrial cytochrome c oxidase genes segregate with late-onset Alzheimer disease. *Proceedings of the National Academy of Sciences of the United States of America*.

[14] Schuessel K., Frey C., Jourdan C. (2006). Aging sensitizes toward ROS formation and lipid peroxidation in PS1M146L transgenic mice. *Free Radical Biology & Medicine*.

[15] Strazielle C., Jazi R., Verdier Y., Qian S., Lalonde R. (2009). Regional brain metabolism with cytochrome c oxidase histochemistry in a PS1/A246E mouse model of autosomal dominant Alzheimer's disease: correlations with behavior and oxidative stress. *Neurochemistry International*.

[16] Moreira P. I., Carvalho C., Zhu X., Smith M. A., Perry G. (2010). Mitochondrial dysfunction is a trigger of Alzheimer's disease pathophysiology. *Biochimica et Biophysica Acta*.

[17] Wang X., Wang W., Li L., Perry G., Lee H., Zhu X. (2014). Oxidative stress and mitochondrial dysfunction in Alzheimer's disease. *Biochimica et Biophysica Acta*.

[18] Cecchi C., Fiorillo C., Sorbi S. (2002). Oxidative stress and reduced antioxidant defenses in peripheral cells from familial Alzheimer's patients. *Free Radical Biology & Medicine*.

[19] Toussaint O., Medrano E. E., von Zglinicki T. (2000). Cellular and molecular mechanisms of stress-induced premature senescence (SIPS) of human diploid fibroblasts and melanocytes. *Experimental Gerontology*.

[20] Kang H. T., Lee K. B., Kim S. Y., Choi H. R., Park S. C. (2011). Autophagy impairment induces premature senescence in primary human fibroblasts. *PLoS One*.

[21] Brunk U. T., Terman A. (2002). The mitochondrial-lysosomal axis theory of aging: accumulation of damaged mitochondria as a result of imperfect autophagocytosis. *European Journal of Biochemistry*.

[22] Yamamoto A., Tagawa Y., Yoshimori T., Moriyama Y., Masaki R., Tashiro Y. (1998). Bafilomycin A1 prevents maturation of autophagic vacuoles by inhibiting fusion between autophagosomes and lysosomes in rat hepatoma cell line, H-4-II-E cells. *Cell Structure and Function*.

[23] Lee J., Giordano S., Zhang J. (2012). Autophagy, mitochondria and oxidative stress: cross-talk and redox signalling. *The Biochemical Journal*.

[24] Vegh C., Stokes K., Ma D. (2019). A bird’s-eye view of the multiple biochemical mechanisms that propel pathology of Alzheimer’s disease: recent advances and mechanistic perspectives on how to halt the disease progression targeting multiple pathways. *Journal of Alzheimer's Disease*.

[25] Lee J.-H., Yu W. H., Kumar A. (2010). Lysosomal proteolysis and autophagy require presenilin 1 and are disrupted by Alzheimer-related PS1 mutations. *Cell*.

[26] Muthukumaran K., Leahy S., Harrison K. (2014). Orally delivered water soluble coenzyme Q10 (Ubisol-Q10) blocks on-going neurodegeneration in rats exposed to paraquat: potential for therapeutic application in Parkinson’s disease. *BMC Neuroscience*.

[27] Naderi J., Lopez C., Pandey S. (2006). Chronically increased oxidative stress in fibroblasts from Alzheimer’s disease patients causes early senescence and renders resistance to apoptosis by oxidative stress. *Mechanisms of Ageing and Development*.

[28] Somayajulu M., McCarthy S., Hung M., Sikorska M., Borowy-Borowski H., Pandey S. (2005). Role of mitochondria in neuronal cell death induced by oxidative stress; neuroprotection by coenzyme Q10. *Neurobiology of Disease*.

[29] Vasilevskaya I. A., Selvakumaran M., Roberts D., O’Dwyer P. J. (2016). JNK1 inhibition attenuates hypoxia-induced autophagy and sensitizes to chemotherapy. *Molecular Cancer Research*.

[30] Bennett B. L., Sasaki D. T., Murray B. W. (2001). SP600125, an anthrapyrazolone inhibitor of Jun N-terminal kinase. *Proceedings of the National Academy of Sciences*.

[31] Yoshii S. R., Mizushima N. (2017). Monitoring and measuring autophagy. *International Journal of Molecular Sciences*.

[32] Sandhu J. K., Pandey S., Ribecco-Lutkiewicz M. (2003). Molecular mechanisms of glutamate neurotoxicity in mixed cultures of NT2-derived neurons and astrocytes: protective effects of coenzyme Q10. *Journal of Neuroscience Research*.

[33] Abate M., Festa A., Falco M. (2019). Mitochondria as playmakers of apoptosis, autophagy and senescence. *Seminars in Cell & Developmental Biology*.

[34] Naderi J., Somayajulu-Nitu M., Mukerji A. (2006). Water-soluble formulation of coenzyme Q10 inhibits Bax-induced destabilization of mitochondria in mammalian cells. *Apoptosis*.

